# Bioactive steroids from seed germination supporting fungus (*Ceratobasidium* GS2) of the terrestrial orchid *Gymnadenia conopsea*

**DOI:** 10.1080/21501203.2023.2254893

**Published:** 2024-01-02

**Authors:** Lixin Shi, Zeyu Zhao, Luna Yang, Gang Ding, Xiaoke Xing

**Affiliations:** Key Laboratory of Bioactive Substances and Resources Utilization of Chinese Herbal Medicine, Ministry of Education, Institute of Medicinal Plant Development, Chinese Academy of Medical Sciences and Peking Union Medical College, Beijing, China

**Keywords:** *Gymnadenia conopsea*, orchid mycorrhizal fungi, *Ceratobasidium* sp., steroids, biologic activity, protocorm differentiation

## Abstract

Almost all orchids rely on mycorrhizal fungus to support their seed germination. To date, the effect of active components in mycorrhizal fungus on orchid seed germination largely remains unknown. In this study, we aimed to investigate the impact of active components found in mycorrhizal fungus on orchid seed germination. Specifically, we focused on a terrestrial orchid *Gymnadenia conopsea* and its host-specific seed germination supporting fungus *Ceratobasidium* GS2. In total, several steroids (**1**–**7**) were isolated from this fungus. Notably, compounds **1**, **2**, **4**, and **5** exhibited significant enhancements in protocorm volume. Moreover, compounds **1**–**6** demonstrated strong promotion of protocorm differentiation. These findings suggest that steroids may play a crucial role in the symbiotic germination of *G. conopsea* seeds. Future studies should continue to explore the specific mechanisms through which these steroids exert their effects, contributing to our understanding of orchid biology and mycorrhizal interaction.

## 1. Introduction

Almost all orchid plants are highly dependent on orchid mycorrhizal fungi (OMF) to support their seed germination and early seedling development, due to their tiny seeds containing no endosperm. Mycorrhizal fungi have been regarded as one of the most important factors affecting the occurring and distribution of orchids (McCormick and Jacquemyn 2013; Pecoraro et al. 2018). Based on the natural mycorrhizal symbiosis characteristic, isolation and selection of compatible fungi and further use in seed symbiotic germination have been proven as an effective way to realise population recovery and conservation of rare and endangered orchids (Shao et al. 2022).

*Gymnadenia conopsea* (L.) R. Br. is a perennial terrestrial orchid that is distributed from 200 to 4,700 m altitude throughout Northern Europe and Asian countries, including Nepal, Japan, Korea, and China (Meekers et al. 2012). In China, the tuber of *G. conopsea* had long been used as traditional Chinese herb medicine, Tibetan medicine, and Mongolian medicine to cure kidney asthenia, cough, dyspnoea induced by lung asthenia, etc. In addition to its medicinal utilisation, *G. conopsea* is also used as a traditional health food in China due to its excellent effect on invigorating the spleen, nourishing the lungs, and regenerating body fluid (Shang et al. 2017). However, to date, *G. conopsea* cannot be artificially cultivated, and habitat loss, increasing commercial demand, and overgrazing have led to a dramatic decline in wild populations of *G. conopsea* in China. As a result, *G. conopsea* has been listed as the class II protected plant in the List of National Key Protected Wild Plants of China in 2021.

In the former research, a fungal strain, *Ceratobasidium* GS2 (NCBI accession no: OK655751.1) was isolated from the roots of *G. conopsea* and shown to promote seed germination and early seedling development under both *in vitro* and *in situ* conditions and displayed potential utilisation in population recovery and artificial production of *G. conopsea* (Gao et al. 2020; Jiang et al. 2022). To date, although it has been well understood that orchid seed germination needs the compatible mycorrhizal fungus to supply nutrients, there are a number of questions associated with orchid mycorrhizal symbiosis not yet fully elucidated. A key one in this regard is how seed germination is promoted by mycorrhizal fungi. Recent research revealed a substantial metabolomic alteration involved in the orchid-OMF interactions (Ghirardo et al. 2020). However, the effect of potentially bioactive molecules from the fungi on the process of symbiotic germination largely remains unknown.

As a class of vital biomolecules, steroids are essential components of fungal membranes and act to maintain fluidity and integrality, and also act as signalling molecules, growth factors to regulate sexual reproduction (Granado et al. 1995; Volkman 2003; Kuhn et al. 2010). In addition, steroids were also known as biosynthetic precursors of brassinolide, a group of steroid phytohormones that regulate diverse processes such as plant growth, development, and stress responses (Vriet et al. 2013). Till now, whether steroids in OMF play a role in the orchid seed symbiotic germination has never been reported. The aim of this research is to isolate and identify bioactive molecules from the *G. conopsea* seed germination-supporting fungus *Ceratobasidium* GS2, and further test their possible functions in the process of symbiotic germination.

## 2. Materials and methods

### 2.1. General experimental procedures

NMR spectra (^1^H: 600 MHz, ^13^C: 150 MHz) were obtained on a Bruker 600 spectrometer. Semi-preparative HPLC separation was performed on a Shimadzu LC-6AD instrument packed with a YMC-Pack ODS-A column. Sephadex LH-20 and silica gel were purchased from Pharmacia (Biotech, Sweden) and Qingdao Marine Chemical Plant (Qingdao, China), respectively. The reagents of HPLC grade (Tedia Company, Fairfield, OH, USA) were used.

### 2.2. Fungal material

The fungal strain *Ceratobasidium* GS2 (NCBI accession no.: OK655751.1) was originally isolated from the roots of *G*. *conopsea* (Gao et al. 2020) and then deposited in the Mycological Herbarium of the Institute of Microbiology, Chinese Academy of Sciences (accession number CGMCC no. 16089). The fermentation procedure was described in previous work (Shi et al. 2022).

### 2.3. Extraction and isolation

*Ceratobasidium* GS2 was grown on PDA plates at 25 °C for 7 days. Then the fresh mycelium was inoculated into autoclaved solid medium containing rice (60.0 g) and distilled water (80 mL) in Fernbach flasks (500 mL) for further fermentation at 25 °C for 30 days.

Using ethyl acetate extracted the fermented rice substrate three times to afford 100 g of crude extract by evaporating the organic solvent under a vacuum. The crude extract was separated by a silica gel column chromatography (CC) eluted with petroleum ether-acetone (100:1, 50:1, 25:1, 15:1, 5:1, 2:1, and 0:1, v/v) to obtain seven fractions (Fr.1 to Fr.7). Fr.2 (2.43 g) was recrystallised to afford **1** (334 mg). Fr.3 (1.23 g) was further separated by Sephadex LH-20 (CH_2_Cl_2_/MeOH, v/v, 1:1) to yield five subfractions (Fr.3.1–Fr.3.5). Fr.3.2 (425 mg) was recrystallised and then purified by semipreparative HPLC (90%–100% CH_3_OH-H_2_O for 30 min) to obtain **2** (9.5 mg, *t*_R_ 30 min). Fr.4 (647 mg) was separated by a silica gel CC eluted with petroleum ether-acetone (100:1, 50:1, 30:1, 20:1, 15:1, 10:1, 5:1, and 0:1, v/v) to get 5 subfractions (Fr.4.1–Fr.4.5). Fr.4.3 (91 mg) was purified by semipreparative HPLC (94%–100% CH_3_OH-H_2_O for 30 min, v/v, 2 mL/min) to afford **3** (41 mg, *t*_R_ 23.8 min), **4** (3 mg, *t*_R_ 44 min), **5** (3.5 mg, *t*_R_ 37 min), **6** (2.9 mg, *t*_R_ 31 min), and **7** (2.1 mg, *t*_R_ 29 min).

*β*-sitosterol (**1**): ^1^H-NMR (CDCl_3_, 600 MHz) *δ*_H_ = 5.35 (H, m, H-6), 3.52 (H, m, H-3), 0.69 (3 H, s, H-18), 1.01 (3 H, s, H-19), 0.92 (3 H, d, *J* = 6.6 Hz, H-21), 0.83 (3 H, d, *J* = 6.6 Hz, H-26), 0.81 (3 H, d, *J* = 6.6 Hz, H-27), 0.84 (3 H, t, *J* = 7.2 Hz, H-29). ^13^C-NMR (CDCl_3_, 150 MHz) *δ*_c_ = 140.7 (C-5), 121.7 (C-6), 71.8 (C-3), 56.7 (C-14), 56.0 (C-17), 50.1 (C-9), 45.8 (C-24), 42.3 (C-4), 42.3 (C-13), 39.7 (C-12), 37.2 (C-1), 36.5 (C-10), 36.1 (C-20), 33.9 (C-22), 31.9 (C-7), 31.9 (C-8), 31.6 (C-2), 29.1 (C-25), 28.3 (C-16), 26.0 (C-23), 24.3 (C-15), 23.0 (C-28), 21.1 (C-11), 19.8 (C-27), 19.4 (C-19), 19.0 (C-26), 18.8 (C-21), 12.0 (C-29),11.9 (C-18). The data were consistent with the literature (Wang et al. 2009).

Stigmast-4-ene-6*β*-ol-3-one (**2**): ^1^H-NMR (CDCl_3_, 600 MHz) *δ*_H_ = 5.81 (H, d, *J* = 1.2 Hz, H-4), 4.35 (H, t, *J* = 7.2 Hz, H-6), 0.74 (3 H, s, H-18), 1.37 (3 H, s, H-19), 0.92 (3 H, d, *J* = 6.6 Hz, H-21), 0.83 (3 H, d, *J* = 6.6 Hz, H-26), 0.81 (3 H, d, *J* = 6.6 Hz, H-27), 0.84 (3 H, t, *J* = 7.2 Hz, H-29). ^13^C-NMR (CDCl_3_, 150 MHz) *δ*_c_ = 200.6 (C-3), 168.5 (C-5), 126.3 (C-4), 73.3 (C-6), 56.0 (C-17), 55.8 (C-14), 53.6 (C-9), 45.8 (C-24), 42.5 (C-13), 39.6 (C-12), 38.5 (C-7), 38.0 (C-10), 37.1 (C-1), 36.1 (C-20), 34.3 (C-2), 33.8 (C-22), 29.7 (C-8), 29.1 (C-25), 28.2 (C-16), 26.0 (C-23), 24.1 (C-15), 23.0 (C-28), 20.9 (C-11), 19.8 (C-26), 19.5 (C-19), 19.0 (C-27), 18.7 (C-21), 12.0 (C-29),12.0 (C-18). The data were consistent with the literature (Wang et al. 2009).

Stigmast-4-ene-6*α*-ol-3-one (**3**): ^1^H-NMR (CDCl_3_, 600 MHz) *δ*_H_ = 6.17 (H, d, *J* = 1.2 Hz, H-4), 4.33 (H, m, H-6), 0.70 (3 H, s, H-18), 1.18 (3 H, s, H-19), 0.91 (3 H, d, *J* = 6.6 Hz, H-21), 0.83 (3 H, d, *J* = 6.6 Hz, H-26), 0.81 (3 H, d, *J* = 6.6 Hz, H-27), 0.84 (3 H, t, *J* = 7.2 Hz, H-29). ^13^C-NMR (CDCl_3_, 150 MHz) *δ*_c_ = 199.5 (C-3), 171.5 (C-5), 119.6 (C-4), 68.7 (C-6), 55.9 (C-17), 55.5 (C-14), 53.7 (C-9), 45.8 (C-24), 42.4 (C-13), 41.5 (C-7), 39.4 (C-12), 39.0 (C-10), 36.3 (C-1), 36.1 (C-20), 34.1 (C-2), 33.8 (C-22), 33.8 (C-8), 29.1 (C-25), 28.1 (C-16), 26.0 (C-23), 24.2 (C-15), 23.0 (C-28), 21.0 (C-11), 19.8 (C-26), 19.0 (C-27), 18.7 (C-21), 18.3 (C-19), 12.0 (C-28), 11.9 (C-18). The data were consistent with the literature (Wang et al. 2009).

*β*-Hydroxystigmast-5-en-7-one (**4**): ^1^H-NMR (CDCl_3_, 600 MHz) *δ*_H_ = 3.68 (H, tt, *J* = 4.8, 11.4 Hz H-3), 5.69 (H, d, *J* = 1.8 Hz, H-6), 0.68 (3 H, s, H-18), 1.20 (3 H, s, H-19), 0.92 (3 H, d, *J* = 6.6 Hz, H-21), 0.83 (3 H, d, *J* = 6.6 Hz, H-26), 0.81 (3 H, d, *J* = 6.6 Hz, H-27), 0.84 (3 H, t, *J* = 7.2 Hz, H-29). ^13^C-NMR (CDCl_3_, 150 MHz) *δ*_c_ = 202.4 (C-7), 165.1 (C-5), 70.5 (C-3), 54.6 (C-17), 49.9 (C-9), 49.9 (C-14), 45.8 (C-24), 45.4 (C-8), 43.1 (C-13), 41.8 (C-4), 38.7 (C-10), 38.3 (C-12), 36.3 (C-1), 36.1 (C-20), 33.9 (C-22), 31.2 (C-2), 29.1 (C-25), 28.6 (C-16), 26.3 (C-15), 26.0 (C-23), 23.0 (C-28), 21.2 (C-11), 19.8 (C-26), 19.0 (C-27), 18.9 (C-21), 17.3 (C-19), 12.0 (C-29), 12.0 (C-18). The data were consistent with the literature (Guerriero et al. 1993).

*β*-hydroxy-stigmasta-4,22-diene-3-one (**5**): ^1^H-NMR (CDCl_3_, 600 MHz) *δ*_H_ = 6.10 (H, s, H-4), 5.07 (H, dd, *J* = 15, 8.8 Hz, H-23), 4.95 (H, dd, *J* = 15, 8.8 Hz, H-22), 4.26 (H, m, H-6), 1.11 (3 H, s, H-19), 0.95 (3 H, d, *J* = 6.6 Hz, H-21), 0.84 (3 H, d, *J* = 6.6 Hz, H-29), 0.78 (3 H, d, *J* = 6.6 Hz, H-26), 0.73 (3 H, d, *J* = 6.6 Hz, H-27), 0.66 (3 H, s, H-18). ^13^C-NMR (CDCl_3_, 150 MHz) *δ*_c_ = 199.7 (C-3), 171.6 (C-5), 138.0 (C-22), 129.6 (C-23), 119.7 (C-4), 68.7 (C-6), 55.8 (C-14), 55.7 (C-17), 53.8 (C-9), 51.3 (C-24), 42.3 (C-13), 41.4 (C-20), 40.5 (C-12), 39.3 (C-7), 39.1 (C-10), 36.3 (C-1), 34.1 (C-2), 33.8 (C-25), 31.9 (C-8), 28.9 (C-16), 25.4 (C-28), 24.3 (C-15), 21.2 (C-21), 21.2 (C-11), 21.0 (C-26), 19.0 (C-27), 18.3 (C-19), 12.3 (C-18), 12.1 (C-29). The data were consistent with the literature (Kontiza et al. 2006).

*E*-7*α*-methoxy-5*α*,6*α*-epoxyergosta-8(14),22-dien-3*β*-ol (**6**): ^1^H-NMR (CDCl_3_, 600 MHz) *δ*_H_ = 3.93 (H, m, H-3), 3.20 (H, d, *J* = 3.0 Hz, H-6), 4.15 (H, m, H-7), 0.83 (3 H, s, H-18), 0.78 (3 H, s, H-19), 1.01 (3 H, d, *J* = 6.6 Hz, H-21), 5.19 (H, m, H-22), 5.19 (H, m, H-22), 0.83 (3 H, d, *J* = 6.6 Hz, H-26), 0.81 (3 H, d, *J* = 6.6 Hz, H-27), 0.89 (3 H, d, *J* = 6.6 Hz, H-28), 3.25 (3 H, s, H-29), 3.49 (H, s, 3-OH). ^13^C-NMR (CDCl_3_, 150 MHz) *δ*_c_ = 153.3 (C-14), 135.4 (C-22), 132.1 (C-23), 122.5 (C-8), 72.6 (C-7), 68.8 (C-3), 65.2 (C-5), 58.5 (C-6), 56.7 (C-17), 54.6 (C-29), 43.1 (C-13), 42.8 (C-24), 40.2 (C-9), 39.6 (C-4), 39.3 (C-20), 36.5 (C-12), 35.9 (C-10), 33.1 (C-25), 32.1 (C-1), 31.1 (C-2), 27.2 (C-16), 24.9 (C-15), 21.2 (C-21), 20.0 (C-26), 19.7 (C-27), 19.2 (C-11), 18.2 (C-18), 17.6 (C-28), 16.5 (C-19). The data were consistent with the literature (Gao et al. 2010).

*β*-acteoxy-(22*E*,24*R*)-24-methyl-5*α*-cholest-7,22-diene-5,6*β*-diol (**7**): ^1^H-NMR (CDCl_3_, 600 MHz) *δ*_H_ = 5.14 (H, m, H-3), 3.60 (H, d, *J* = 4.8 Hz, H-6), 5.34 (H, dd, *J* = 4.8, 2.2 Hz, H-7), 0.59 (3 H, s, H-18), 1.09 (3 H, s, H-19), 1.02 (3 H, d, *J* = 6.6 Hz, H-21), 5.20 (H, m, H-22), 5.20 (H, m, H-23), 0.83 (3 H, d, *J* = 6.6 Hz, H-26), 0.83 (3 H, d, *J* = 6.6 Hz, H-27), 0.91 (3 H, d, *J* = 6.6 Hz, H-28), 2.04 (3 H, s, Ac). ^13^C-NMR (CDCl_3_, 150 MHz) *δ*_c_ = 144.1 (C-8), 135.4 (C-22), 132.1 (C-23), 117.4 (C-7), 75.7 (C-5), 73.7 (C-6), 71.0 (C-3), 55.9 (C-17), 54.7 (C-14), 43.7 (C-13), 43.2 (C-9), 42.8 (C-24), 40.5 (C-20), 39.1 (C-12), 37.1 (C-10), 35.7 (C-4), 33.1 (C-25), 32.6 (C-1), 27.9 (C-2), 26.9 (C-16), 22.9 (C-15), 21.9 (C-11), 21.1 (C-21), 20.0 (C-26), 19.6 (C-27), 18.7 (C-19), 17.6 (C-28), 12.3 (C-18), 170.7/21.5 (Ac). The data were consistent with the literature (Huang et al. 2001).

### 2.4. Seedling growth test

The sample solutions were prepared in DMSO, ranging from 0.001 mg/mL to 2 mg/mL. 100 µL sample was coated on solid media in 9 cm Petri dishes and 100 µL of DMSO was used as a control. The seeds of *G. conopsea* were treated with calcium hypochlorite solutions for 5 min, washed 3 times with sterile distilled water, 70% ethanol for 5 min, washed 3 times with distilled water, and the suspension of seed was poured onto OMA medium (5 g rolled oats +12 g agar/L). And then approximately, 3 mm × 3 mm piece of *Ceratobasidium* GS2 fungus-infected medium was placed on the same OMA medium because the seed could not grow in the absence of the fungus. The dishes were kept in the dark at 25 °C for symbiotic cultures. Morphological changes of the seed and the fungus during seed germination were observed daily under a stereomicroscope. Four developmental stages of *G. conopsea* were defined: Stage 1: Germination; Stage 2: Protocorm formation; Stage 3: Protocorm differentiation; Stage 4: Seedling with emergence of first leaf (Gao et al. 2020). In this study, the percentages of seed germination and protocorm formation were analysed 30 days after sowing (4–6 plates from each treatment) (Wang et al. 2004; Gao et al. 2020). Protocorm differentiation was also analysed at the corresponding developmental stage 60 days after sowing. The statistical significance was considered for *P*-values less than 0.05 in a one-way ANOVA (Xiao et al. 2019). All statistical analyses were performed using GraphPad prism 8.

## 3. Results

### 3.1. Structural identification

**Figure 1.**
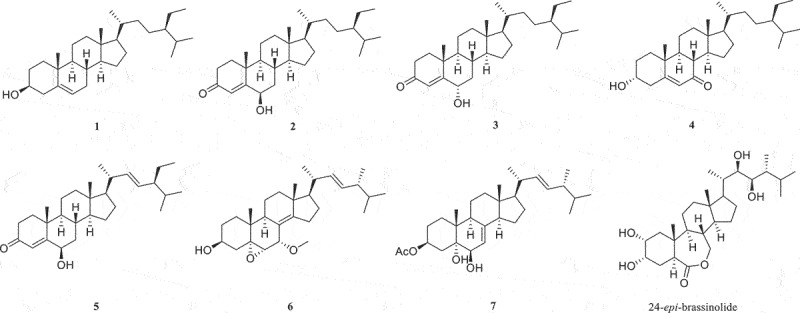
The structures of the compounds **1**–**7** and 24-*epi*-brassinolide.

Seven steroids were identified as *β*-sitosterol (**1**) (Figure S1 and S2), stigmast-4-ene-6*β*-ol-3-one (**2**) (Figure S3 and S4), stigmast-4-ene-6*α*-ol-3-one (**3**) (Figure S5 and S6), 3*β*-hydroxystigmast-5-en-7-one (**4**) (Figure S7 and S8), 6*β*-hydroxy-stigmasta-4,22-diene-3-one (**5**) (Figure S9 and S10), 22*E*-7*α*-methoxy-5*α*,6*α*-epoxyergosta-8(14),22-dien-3*β*-ol (**6**) (Figure S11 and S12), and 3*β*-acteoxy-(22*E*, 24*R*)-24-methyl-5*α*-cholest-7,22-diene-5,6*β* -diol (**7**) (Figure S13 and S14), respectively, based on analysis of NMR data and comparison with literatures (Figure 1).

### 3.2. *Effects of compounds 1–6 on* Ceratobasidium *GS2*

**Figure 2.**
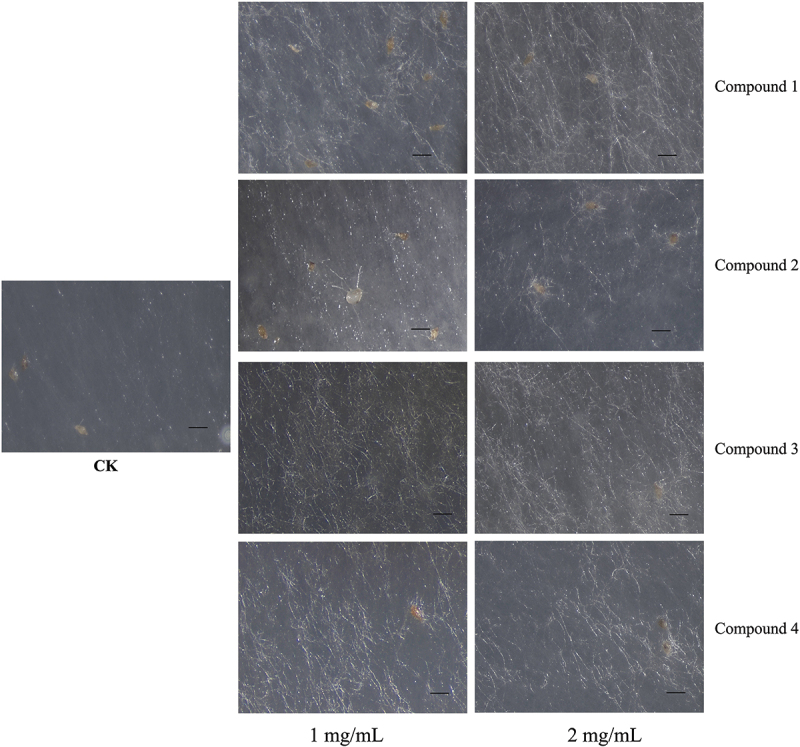
Growth characteristics of *Ceratobasidium* GS2 in control group and treatment groups. Scale bars = 500 μm.

Different concentrations of compounds **1**–**6** (0.001, 0.01, 0.1, 1, and 2 mg/mL) were added exogenously in a medium to observe their effects on the fungus *Ceratobasidium* GS2, respectively. Compound **7** did not test assay due to limited amounts in this report. These results showed that compounds could not affect the radial growth of the fungus as the fungus colony size of treatment and control groups were consistent over time. It was found that compounds **1**–**4** with high concentrations of 1–2 mg/mL could make the fungi growth denser in 20 days (Figure 2). In contrast, the effect was not seen at 0.001–0.1 mg/mL of compounds **1**–**4** or when the fungus was grown in the presence and at all concentrations of compounds **5** and **6** (Figure S15 and S16).

### 3.3. Effects of compounds 1–6 on seed germination

**Figure 3.**
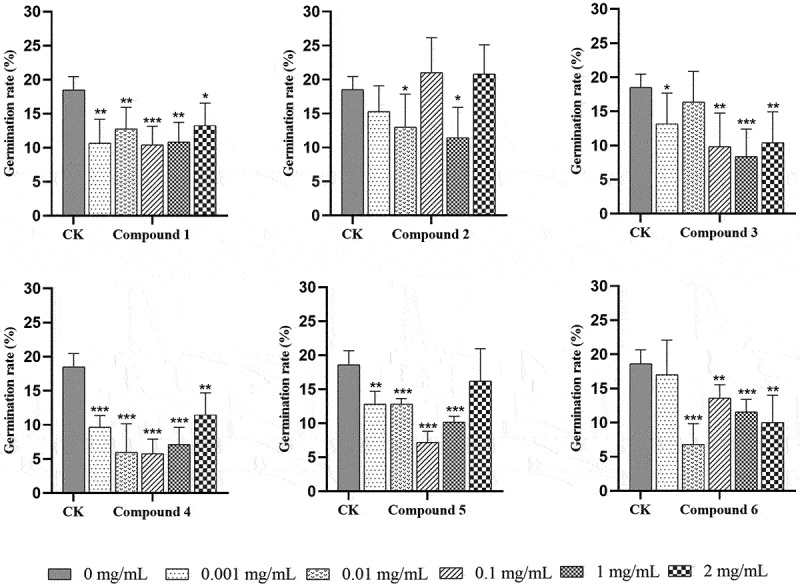
The effects of different treatments on seed germination rate of *Gymnadenia conopsea* after 30 days. The number of *indicates the level of significant difference, *P < 0.05, **P < 0.01, ***P < 0.001.

After 30 days of incubation, seeds in all the treatments germinated and protocorms formatted. The seed germination rate in each packet was calculated, and the seed germination rate of exogenous addition treatments (**1** and **3**–**6**) was lower than that of the control group (17.5%) (Figure 3), in which the seed germination rate of compounds **1** and **4** addition treatments significantly decreased (*P* < 0.05). The lowest rate of seed germination occurred at concentration (0.01–1 mg/mL) based on calculation and visual inspection. There were no significant differences in the compound **2** treatment at 0.1 and 2 mg/mL, though seed germination was promoted slightly, yet seed germination was inhibited in the concentrations of exogenous compound **2** at 0.001, 0.01, and 1 mg/mL compared with the control treatment. Compared with other exogenous steroids addition, compound **4** had a serious inhibitory effect on seed germination with rates less than 10%. The five concentrations of each compound addition treatment differed but without concentration dependence.

### 3.4. Effects of compounds 1–6 on protocorm volume

**Figure 4.**
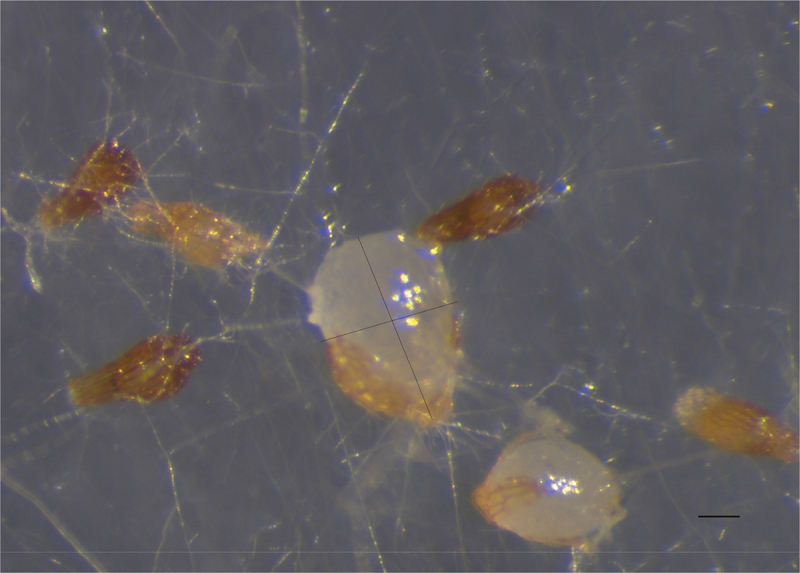
The volume of *Gymnadenia conopsea* protocorm after 30 days’ inoculation. Scale bars = 500 μm.

**Figure 5.**
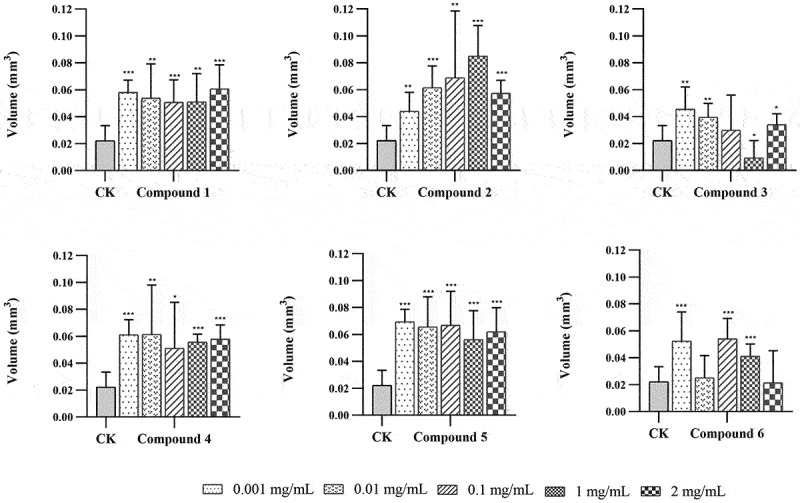
The effects of different treatments on the volume of *Gymnadenia conopsea* protocorm after 30 days. The number of *indicates the level of significant difference, *P < 0.05, **P < 0.01, ***P < 0.001.

After 30 days of culture on the medium, protocorms were harvested at stage 2. The volume of each protocorm was calculated using the equation: volume (mm^3^) = (πlb^2^)/6 where l = length and b = breadth at the widest point (mm) (Hadley and Williamson 1971; Mckendrick et al. 2000) (Figure 4). The majority of seeds germinated only grew to 0.02–0.08 mm^3^. Interestingly, a very small minority grew much larger. Therefore, the 10 greatest protocorms in each treatment were calculated. In germination trials, treatments with compounds **1**, **2**, **4**, and **5** showed a significant promoting effect on protocorm volume in comparison with control (all *P* < 0.01). No significant promoting activity by treatments of compounds **3** and **6** were observed (compound **3**, 0.1 mg/mL; compound **6**, 0.01 mg/mL and 2 mg/mL) (*P* > 0.05) and even a bit of inhibition effect from treatment of compound **3** (1 mg/mL) (*P* < 0.05) was found (Figure 5).

### 3.5. Effects of compounds 1–6 on protocorm differentiation

**Figure 6.**
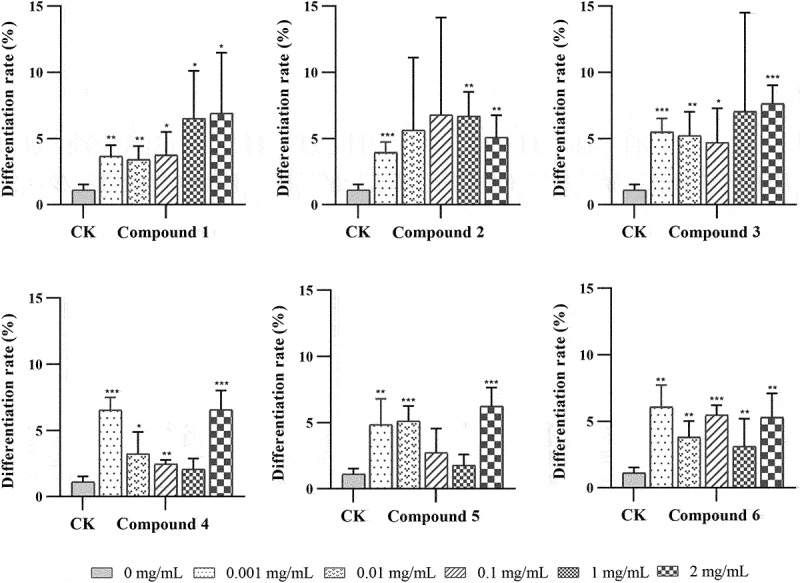
The effects of different treatments on *Gymnadenia conopsea* protocorm differentiation after 60 days. The number of *indicates the level of significant difference, *P < 0.05, **P < 0.01, ***P < 0.001.

After 60 days of culture in OMA, *G. conopsea* seeds developed to stage 3. Most seeds had germinated and presented a significantly higher protocorm differentiation in all treatments (Figure 6). The differentiation ratio of compound **6** at different concentration treatments was significantly higher than the control (*P* < 0.01). For compound **2**, the differentiation ratio increased first and then decreased as the concentration increased. Among the treatments with different concentrations, the 0.1 mg/mL compound **2** treatment displayed the maximal effect on differentiation ratio but showed no significant differences with the control. However, the ratio decreased first and then increased as compounds **3** and **4** concentration increased. Compound **5** at 0.001, 0.01, and 2 mg/mL treatment groups increased and significantly differed from the control (*P* < 0.01). The differentiation ratios of compounds **1**, **3**, **4**, and **6** at 0.1 mg/mL treatment were significantly higher than those of the control group (*P* < 0.05), and the ratios of compounds **1**, **2**, and **6** at 1 mg/mL treatment were also high with a significant level compared with those of the control group (*P* < 0.05).

## 4. Discussion

Symbiotic germination of orchid seeds involves the dual process of seed self-development and mutualistic interaction with their mycorrhizal fungi. Thus, the process is quite complex physiologically and ecologically. A variety of studies have revealed that the secondary metabolites of plant and fungus were important factors affecting seed germination and were also critical for the establishment of mycorrhizal symbiosis, but up to date no small molecules were found related to seed germination (Akiyama et al. 2005; Yoneyama et al. 2007; Oldroyd 2013; Sun et al. 2015; Hou et al. 2016). This study isolated a series of steroids from the medicinal orchid *G. conopsea* seed germination-supporting fungus *Ceratobasidium* GS2 for the first time and revealed that high concentrations of compounds **1**–**4** were beneficial for fungal growth. In addition, exogenous steroids could also be confirmed to promote protocorm development and differentiation.

This study examined the effect of the extract on the growth of *Ceratobasidium* GS2 itself. The addition of high concentrations of steroids resulted in denser growth of hypha, which indicates that these compounds have a stimulating effect on their growth and reproduction. This result is consistent to with the former findings that the autologous extract of fungi can affect their growth. For example, furanoids isolated from *Ceratobasidium* sp. could inhibit their growth (Shi et al. 2022). Fungi have different reactions to autologous extract; however, the mechanisms still need to be unexplored.

Steroids are a member of bioactive metabolites from different fungi. Most of steroids are ergosterol metabolites including cholesterol, stigmasterol, and their derivatives (Zhabinskii et al. 2022). Among them, ergosterol is mainly involved in regulating of membrane fluidity and structure, and performing immunological functions (Rodrigues 2018). *β*-sitosterol (**1**) (SIT) is a bioactive stigmasterol with various biological roles such as anxiolytic and sedative, analgesic, immunomodulatory, antimicrobial, anticancer, anti-inflammatory, lipid-lowering, hepatoprotective, protective effects against respiratory diseases, and wound healing, antioxidant and anti-diabetic activities (Lopez-Rubalcava et al. 2006; Ododo et al. 2016; Paniagua-Perez et al. 2017; Abdou et al. 2019; Babu and Jayaraman 2020). In addition, ergosterol and its analogues also exhibited excellent antibacterial activity (Yenn et al. 2012). Interestingly, previous results indicated that ergosterol isolated from the methanol extract of *Armillaria mellea* could accelerate the vegetative propagation of *Gastrodia elata* protocorms (Guo et al. 1996). Consistent with the former finding, steroids isolated in this research also exhibited enhancing protocorm development. These results further implied that steroids might play a critical role in symbiotic seed germination of the medicinal orchid *G. conopsea*.

Almost all orchids rely on a symbiotic relationship with fungi for seed germination and protocorm development (Zhao et al. 2021; Tian et al. 2022). The host plant obtains nutrient by digestion of the penetrated hypha in cortex cells (Zhao et al. 2013; Yang et al. 2018). The protocorm formed at the beginning of seed germination is considered as a unique structure, which is designed to establish a symbiotic association with mycorrhizal fungi (Rasmussen 1990). In this research, the abilities of steroids to promote the development of protocorms make them respond and prepare to interact with mycorrhizal fungi (Yeung 2017). Therefore, steroids might be a kind of active ingredients that promote the growth of protocorms. The protocorm will gradually differentiate to form a shoot apical meristem and further develop into a seedling (Sussex 1989). The higher protocorm differentiation rate induced by steroids may link to a higher survival chance of orchids in their natural habitats (Hossain et al. 2013).

Early research had revealed that steroids are the biosynthetic precursors of brassinolides (Zhao and Li 2012; Roh et al. 2020), plant steroid hormones. Consistently with their role as precursors for the production of brassinolide, sterols possess similar biological functions as brassinolides. It has been reported that BRs might enhance orchid protocorm elongation by regulating auxin transport through an F-actin-mediated mechanism (Novak et al. 2018), which implied that the steroids, the potent precursors of brassinolides, may be involved in hair outgrowth and developing protocorms. However, more studies are needed to investigate understanding of the regulatory mechanism in orchid development.

## 5. Conclusion

Seven steroid derivatives (**1**–**7**) were first purified from the *G. conopsea* seed germination supporting fungus *Ceratobasidium* GS2, in which compounds **1**, **2**, **4**, and **5** could significantly increase the protocorm volume, and the protocorm differentiation rates were also promoted by compounds **1**–**6**. The results will contribute to understanding the symbiotic molecular mechanism and further utilisation and development of steroid analogs from symbiotic germination supporting fungi.

## Supplementary Material

Supplemental Material

## References

[cit0001] Abdou EM, Fayed MA, Helal D, Ahmed KA. 2019. Assessment of the hepatoprotective effect of developed lipid-polymer hybrid nanoparticles (LPHNPs) encapsulating naturally extracted *β*-sitosterol against CCl_4_ induced hepatotoxicity in rats. Sci Rep. 9(1):19779. doi: 10.1038/s41598-019-56320-2. 31875004 PMC6930297

[cit0002] Akiyama K, Matsuzaki K, Hayashi H. 2005. Plant sesquiterpenes induce hyphal branching in arbuscular mycorrhizal fungi. Nature. 435(7043):824–827. doi:10.1038/nature03608. 15944706

[cit0003] Babu S, Jayaraman S. 2020. An update on beta-sitosterol: a potential herbal nutraceutical for diabetic management. Biomed Pharmacother. 131:110702. doi:10.1016/j.biopha.2020.110702. 32882583

[cit0004] Gao H, Hong K, Chen GD, Wang CX, Tang JS, Yu Y, Jiang MM, Li MM, Wang NL, Yao XS. 2010. New oxidized sterols from *Aspergillus awamori* and the endo-boat conformation adopted by the cyclohexene oxide system. Magn Reson Chem. 48(1):38–43. doi:10.1002/mrc.2536. 19877128

[cit0005] Gao Y, Zhao Z, Li J, Liu N, Jacquemyn H, Guo S, Xing X. 2020. Do fungal associates of co-occurring orchids promote seed germination of the widespread orchid species *Gymnadenia conopsea*? Mycorrhiza. 30(2–3):221–228. doi:10.1007/s00572-020-00943-1. 32146514

[cit0006] Ghirardo A, Fochi V, Lange B, Witting M, Schnitzler JP, Perotto S, Balestrini R. 2020. Metabolomic adjustments in the orchid mycorrhizal fungus *Tulasnella calospora* during symbiosis with *Serapias vomeracea*. New Phytol. 228(6):1939–1952. doi:10.1111/nph.16812. 32668507

[cit0007] Granado J, Felix G, Boller T. 1995. Perception of fungal sterols in plants subnanomolar concentrations of ergosterol elicit extracellular alkalinization in tomato cells. Plant Physiol. 107:485–490. doi:10.1104/pp.107.2.485. 12228375 PMC157151

[cit0008] Guerriero A, D’Ambrosio M, Pietra F. 1993. Pteridines, sterols, and indole derivatives from the lithistid sponge corallistes undulatus of the coral sea. J Nat Prod. 56(11):1962–1970. doi:10.1021/np50101a015.

[cit0009] Guo WJ, Guo SX, Yang JS. 1996. Studies on the fungal metabolites affecting the growth and active components of three traditional Chinese Medicine. [Dissertation]. Beijing: Peking Union Medical College and Chinese Academy of Medical Sciences.

[cit0010] Hadley BG, Williamson B. 1971. Analysis of the post-infection growth stimulus in orchid mycorrhiza. New Phytol. 70(3):445–455. doi:10.1111/j.1469-8137.1971.tb02546.x.

[cit0011] Hossain MM, Kant R, Van PT, Winarto B, Zeng SJ, Teixeira da Silva JA. 2013. The application of biotechnology to orchids. Cait Rev Plant Sci. 32(2):69–139. doi:10.1080/07352689.2012.715984.

[cit0012] Hou SJ, Chen BD, Zhang X. 2016. Signal recognition mechanism in establishing arbuscular mycorrhiza symbiosis. Microbiol China. 43(12):2693–2699. doi: 10.13344/j.microbiol.china.160046.

[cit0013] Huang Y, Dong ZJ, Ji L. 2001. The chemical constituents from basidiocarps of *Sarcodon aspratum*. Acta Bot Yunnan. 23(4):125–128.

[cit0014] Jiang XL, Zhao ZY, Jacquemyn H, Ding G, Ding WL, Xing XK. 2022. Addition of fungal inoculum increases seed germination and protocorm formation in a terrestrial orchid. Global Ecol Conserv. 38:e02235. doi:10.1016/j.gecco.2022.e02235.

[cit0015] Kontiza I, Abatis D, Malakate K, Vagias C, Roussis V. 2006. 3-Keto steroids from the marine organisms *Dendrophyllia cornigera* and *Cymodocea nodosa*. Steroids. 71(2):177–181. doi:10.1016/j.steroids.2005.09.004. 16280145

[cit0016] Kuhn H, Kuster H, Requena N. 2010. Membrane steroid-binding protein 1 induced by a diffusible fungal signal is critical for mycorrhization in *Medicago truncatula*. New Phytol. 185(3):716–733. doi:10.1111/j.1469-8137.2009.03116.x. 20003073

[cit0017] Lopez-Rubalcava C, Pina-Medina B, Estrada-Reyes R, Heinze G, Martinez-Vazquez M. 2006. Anxiolytic-like actions of the hexane extract from leaves of *Annona cherimolia* in two anxiety paradigms: possible involvement of the GABA/benzodiazepine receptor complex. Life Sci. 78(7):730–737. doi:10.1016/j.lfs.2005.05.078. 16122763

[cit0018] McCormick MK, Jacquemyn H. 2013. What constrains the distribution of orchid populations? New Phytol. 202(2):392–400. doi:10.1111/nph.12639.

[cit0019] Mckendrick S, Leake J, Taylor D, Read D. 2000. Symbiotic germination and development of myco-heterotrophic plants in nature: ontogeny of *Corallorhiza trifida* and characterization of its mycorrhizal fungi. New Phytol. 145(3):523–537. doi:10.1046/j.1469-8137.2000.00603.x. 33862904

[cit0020] Meekers T, Hutchings MJ, Honnay O, Jacquemyn H. 2012. Biological flora of the British Isles: *Gymnadenia conopsea s.L*. J Ecol. 100(5):1269–1288. doi:10.1111/j.1365-2745.2012.02006.x.

[cit0021] Novak S, Kalbakji N, Upthegrove K, Neher W, Jones J, de Leon J. 2018. Evidence for brassinosteroid-mediated PAT during germination of *Spathoglottis plicata* (Orchidaceae). Front Plant Sci. 9:1215. doi:10.3389/fpls.2018.01215. 30174682 PMC6107755

[cit0022] Ododo MM, Choudhury MK, Dekebo AH. 2016. Structure elucidation of beta-sitosterol with antibacterial activity from the root bark of *Malva parviflora*. SpringerPlus. 5(1):1210. doi:10.1186/s40064-016-2894-x. 27516948 PMC4967061

[cit0023] Oldroyd G. 2013. Speak, friend, and enter: signalling systems that promote beneficial symbiotic associations in plants. Nat Rev Microbiol. 11(4):252–263. doi:10.1038/nrmicro2990. 23493145

[cit0024] Paniagua-Perez R, Flores-Mondragon G, Reyes-Legorreta C, Herrera-Lopez B, Cervantes-Hernandez I, Madrigal-Santillan O, Morales-Gonzalez JA, Alvarez-Gonzalez I, Madrigal-Bujaidar E. 2017. Evaluation of the anti-inflammatory capacity of *β*-sitosterol in rodent assays. Afr J Tradit Complement Altern Med. 14(1):123–130. doi:10.21010/ajtcam.v14i1.13. 28480389 PMC5411862

[cit0025] Pecoraro L, Caruso T, Cai L, Gupta VK, Liu ZJ. 2018. Fungal networks and orchid distribution: new insights from above- and below-ground analyses of fungal communities. IMA Fungus. 9(1):1–11. doi:10.5598/imafungus.2018.09.01.01. 30018868 PMC6048571

[cit0026] Rasmussen BH. 1990. Cell differentiation and mycorrhizal infection in *Dactylorhiza majalis* (Rchb. f.) Hunt & Summerh. (Orchidaceae) during germination *in vitro*. New Phytol. 116(1):137–147. doi:10.1111/j.1469-8137.1990.tb00519.x.

[cit0027] Rodrigues ML. 2018. The multifunctional fungal ergosterol. mBio. 9(5):e01755–01718. doi:10.1128/mBio.01755-18. PMC6143734 30228244

[cit0028] Roh J, Moon J, Youn JH, Seo C, Park YJ, Kim SK. 2020. Establishment of biosynthetic pathways to generate castasterone as the biologically active brassinosteroid in *Brachypodium distachyon*. J Agric Food Chem. 68(13):3912–3923. doi:10.1021/acs.jafc.9b07963. 32146811

[cit0029] Shang XF, Guo X, Liu Y, Pan H, Miao XL, Zhang JY. 2017. *Gymnadenia conopsea* (L.) R. Br.: a systemic review of the ethnobotany, phytochemistry, and pharmacology of an important Asian folk medicine. Front Pharmacol. 8:24. doi:10.3389/fphar.2017.00024. 28217096 PMC5289989

[cit0030] Shao SC, Luo Y, Jacquemyn H. 2022. Successful reintroduction releases pressure on China’s orchid species. Trends Plant Sci. 27(3):211–213. doi:10.1016/j.tplants.2021.11.018. 34876338

[cit0031] Shi LX, Han L, Zhao ZY, Li Q, Wang YD, Ding G, Xing XK. 2022. Furanoids from the *Gymnadenia conopsea* (Orchidaceae) seed germination supporting fungus *Ceratobasidium* sp. (GS2). Front Microbiol. 13:1037292. doi:10.3389/fmicb.2022.1037292. 36466680 PMC9712750

[cit0032] Sun J, Miller J, Granqvist E, Wiley-Kalil A, Gobbato E, Maillet F, Cottaz S, Samain E, Venkateshwaran M, Fort S, et al. 2015. Activation of symbiosis signaling by arbuscular mycorrhizal fungi in legumes and rice. Plant Cell. 27(3):823–838. doi:10.1105/tpc.114.131326. 25724637 PMC4558648

[cit0033] Sussex LM. 1989. Developmental programming of the shoot kleristem. Cell. 56(2):225–229. doi:10.1016/0092-8674(89)90895-7. 2643476

[cit0034] Tian F, Liao XF, Wang LH, Bai XX, YB Y, Luo ZQ, Yan FX. 2022. Isolation and identification of beneficial orchid mycorrhizal fungi in *Paphiopedilum barbigerum* (Orchidaceae). Plant Signal Behav. 17(1):2005882. doi: 10.1080/15592324.2021.2005882. 34913407 PMC8920121

[cit0035] Volkman JK. 2003. Sterols in microorganisms. Appl Microbiol Biotechnol. 60(5):495–506. doi:10.1007/s00253-002-1172-8. 12536248

[cit0036] Vriet C, Russinova E, Reuzeau C. 2013. From squalene to brassinolide: the steroid metabolic and signaling pathways across the plant kingdom. Mol Plant. 6(6):1738–1757. doi:10.1093/mp/sst096. 23761349

[cit0037] Wang YR, Yu L, Nan ZB, Liu YL. 2004. Vigor tests used to rank seed lot quality and predict field emergence in four forage species. Cropence. 44(2):535–541. doi:10.2135/cropsci2004.5350.

[cit0038] Wang ZJ, Zhao QS, Peng LY, Cheng X. 2009. Study on the chemical constituents of *Callipteris esculenta* (Athyriaceae). Nat Prod Res Dev. 21(6):960–962.

[cit0039] Xiao S, Liu LT, Wang H, Li DX, Bai ZY, Zhang YJ, Sun HC, Zhang K, Li C, Roach T. 2019. Exogenous melatonin accelerates seed germination in cotton (*Gossypium hirsutum* L.). PLoS One. 14(6):e0216575. doi:10.1371/journal.pone.0216575. 31237880 PMC6592504

[cit0040] Yang JW, Ling H, Zhang Y, Zeng X, Guo SX. 2018. Effects of endophytic fungi on seed germination of medicinal plants of Orchidaceae: a review. Mycosystema. 37(1):22–34. doi: 10.13346/j.mycosystema.170198.

[cit0041] Yenn TW, Lee CC, Ibrahim D, Zakaria L. 2012. Enhancement of anti-candidal activity of endophytic fungus *Phomopsis* sp. ED2, isolated from *Orthosiphon stamineus* Benth, by incorporation of host plant extract in culture medium. J Microbiol. 50(4):581–585. doi:10.1007/s12275-012-2083-8. 22923105

[cit0042] Yeung EC. 2017. A perspective on orchid seed and protocorm development. Bot Stud. 58(1):33. doi: 10.1186/s40529-017-0188-4. 28779349 PMC5544657

[cit0043] Yoneyama K, Xie X, Kusumoto D, Sekimoto H, Sugimoto Y, Takeuchi Y, Yoneyama K. 2007. Nitrogen deficiency as well as phosphorus deficiency in sorghum promotes the production and exudation of 5-deoxystrigol, the host recognition signal for arbuscular mycorrhizal fungi and root parasites. Planta. 227(1):125–132. doi:10.1007/s00425-007-0600-5. 17684758

[cit0044] Zhabinskii VN, Drasar P, Khripach VA. 2022. Structure and biological activity of ergostane-type steroids from fungi. Molecules. 27(7):1–53. doi: 10.3390/molecules27072103. PMC9000798 35408501

[cit0045] Zhao BL, Li J. 2012. Regulation of brassinosteroid biosynthesis and inactivation. J Integr Plant Biol. 54(10):746–759. doi:10.1111/j.1744-7909.2012.01168.x. 22963251

[cit0046] Zhao DK, Selosse MA, Wu LM, Luo Y, Shao SM, Ruan YL. 2021. Orchid reintroduction based on seed germination-promoting mycorrhizal fungi derived from protocorms or seedlings. Front Plant Sci. 12:701152. doi:10.3389/fpls.2021.701152. 34276753 PMC8278863

[cit0047] Zhao MM, Zhang G, Zhang DW, Hsiao YY, Guo SX, Roberts D. 2013. Ests analysis reveals putative genes involved in symbiotic seed germination in *Dendrobium officinale*. PLoS One. 8(8):e72705. doi:10.1371/journal.pone.0072705. 23967335 PMC3742586

