# Supplementary material for: Bioactive steroids from seed germination supporting fungus (Ceratobasidium GS2) of the terrestrial orchid Gymnadenia conopsea
Source: Mycology. 2024 Jan 2;14(4):371–80. doi: 10.1080/21501203.2023.2254893 (PMC10769133; doi:10.1080/21501203.2023.2254893)
Supplement: Supplemental Material [file TMYC_A_2254893_SM9385.pdf]

List of Contents

Figure S1. ^{1}H-NMR spectrum of **1** ($CDCl_3$, 600MHz)

Figure S2. ^{13}C-NMR spectrum of **1** ($CDCl_3$, 150MHz)

Figure S3. ^{1}H-NMR spectrum of **2** ($CDCl_3$, 600MHz)

Figure S4. ^{13}C-NMR spectrum of **2** ($CDCl_3$, 150MHz)

Figure S5. ^{1}H-NMR spectrum of **3** ($CDCl_3$, 600MHz)

Figure S6. ^{13}C-NMR spectrum of **3** ($CDCl_3$, 150MHz)

Figure S7. ^{1}H-NMR spectrum of **4** ($CDCl_3$, 600MHz)

Figure S8. ^{13}C-NMR spectrum of **4** ($CDCl_3$, 150MHz)

Figure S9. ^{1}H-NMR spectrum of **5** ($CDCl_3$, 600MHz)

Figure S10. ^{13}C-NMR spectrum of **5** ($CDCl_3$, 150MHz)

Figure S11. ^{1}H-NMR spectrum of **6** ($CDCl_3$, 600MHz)

Figure S12. ^{13}C-NMR spectrum of **6** ($CDCl_3$, 150MHz)

Figure S13. ^{1}H-NMR spectrum of **7** ($CDCl_3$, 600MHz)

Figure S14. ^{13}C-NMR spectrum of **7** ($CDCl_3$, 150MHz)

Figure S15. Growth characteristics of *Ceratobasidium* GS2 in control group and compounds **1-4** treatment. Scale bars=500 μm

Figure S16. Growth characteristics of *Ceratobasidium* GS2 in compounds **5-6** treatment. Scale bars=500 μm

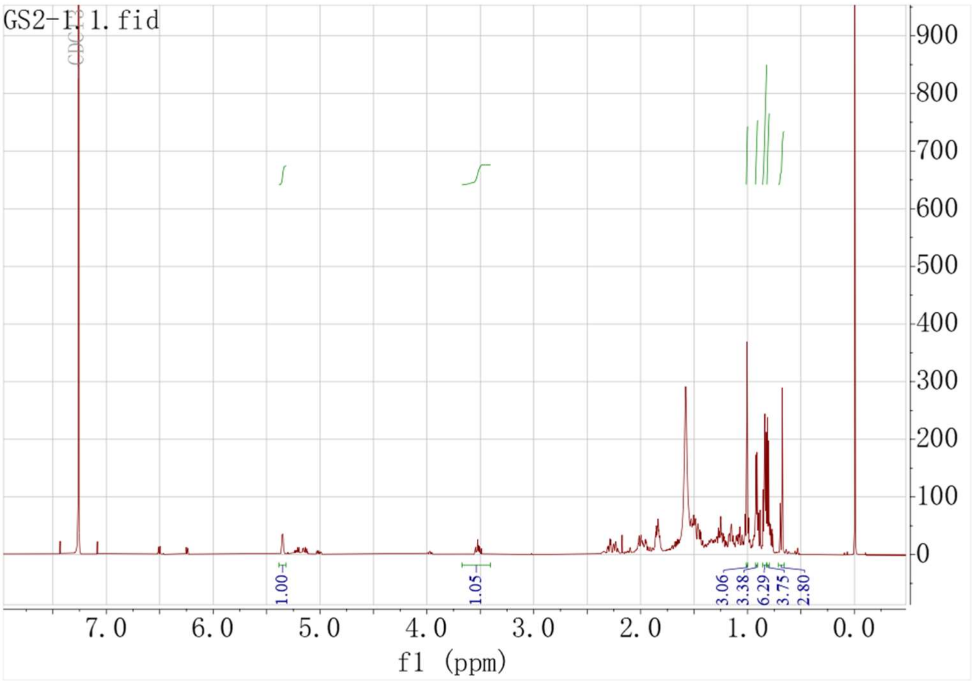

Figure S1. ^{1}H-NMR spectrum of **1** ($CDCl_3$, 600MHz)

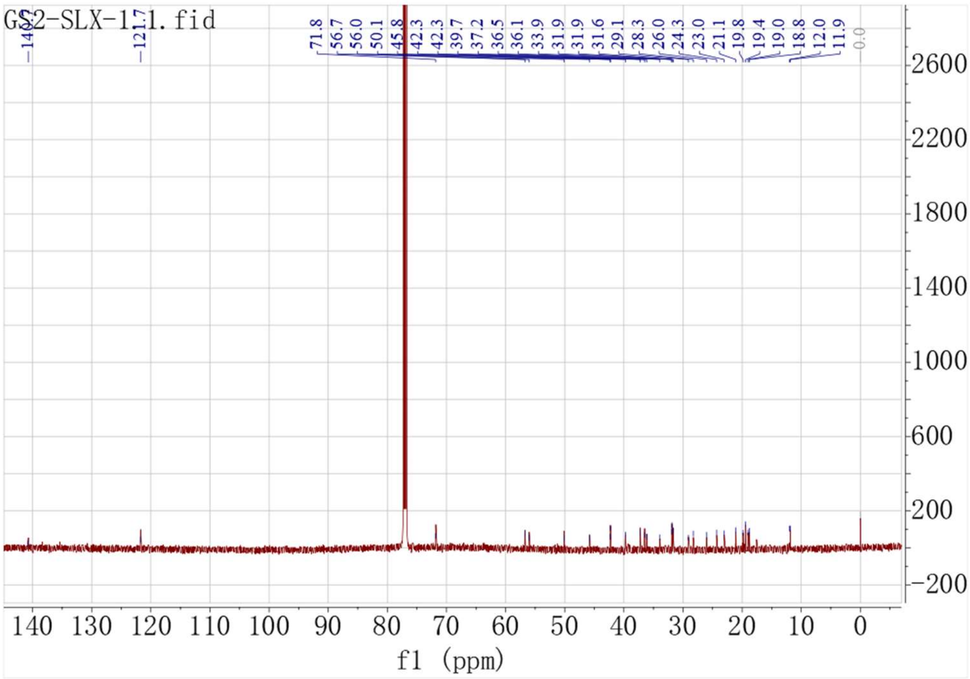

Figure S2. ^{13}C-NMR spectrum of **1** ($CDCl_3$, 150MHz)

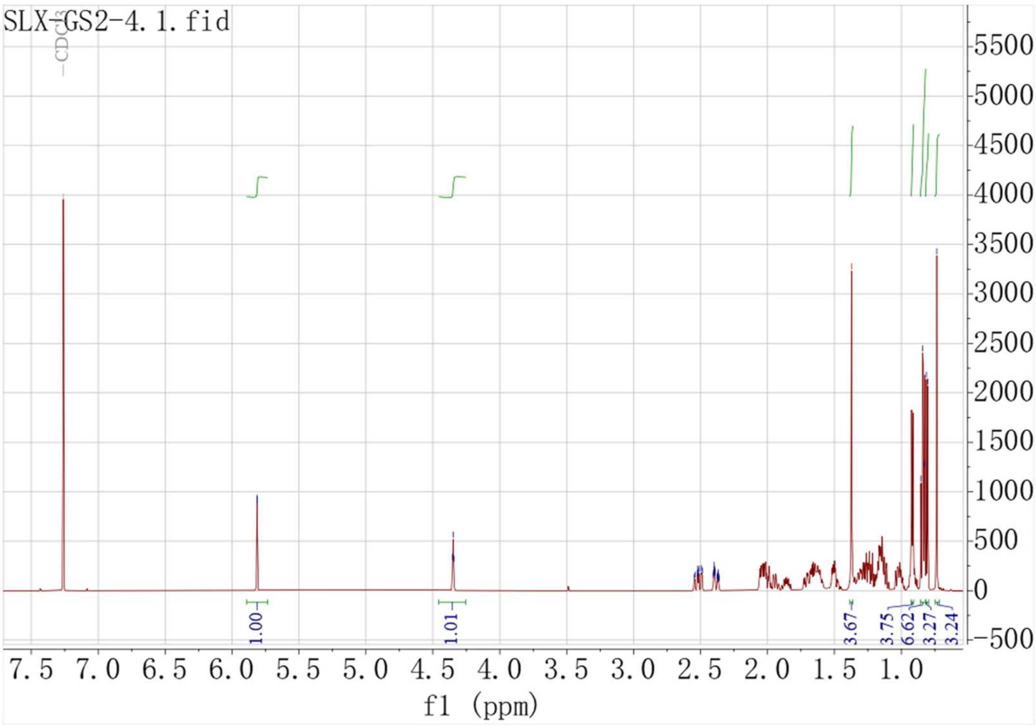

Figure S3. ^{1}H-NMR spectrum of **2** ($CDCl_3$, 600MHz)

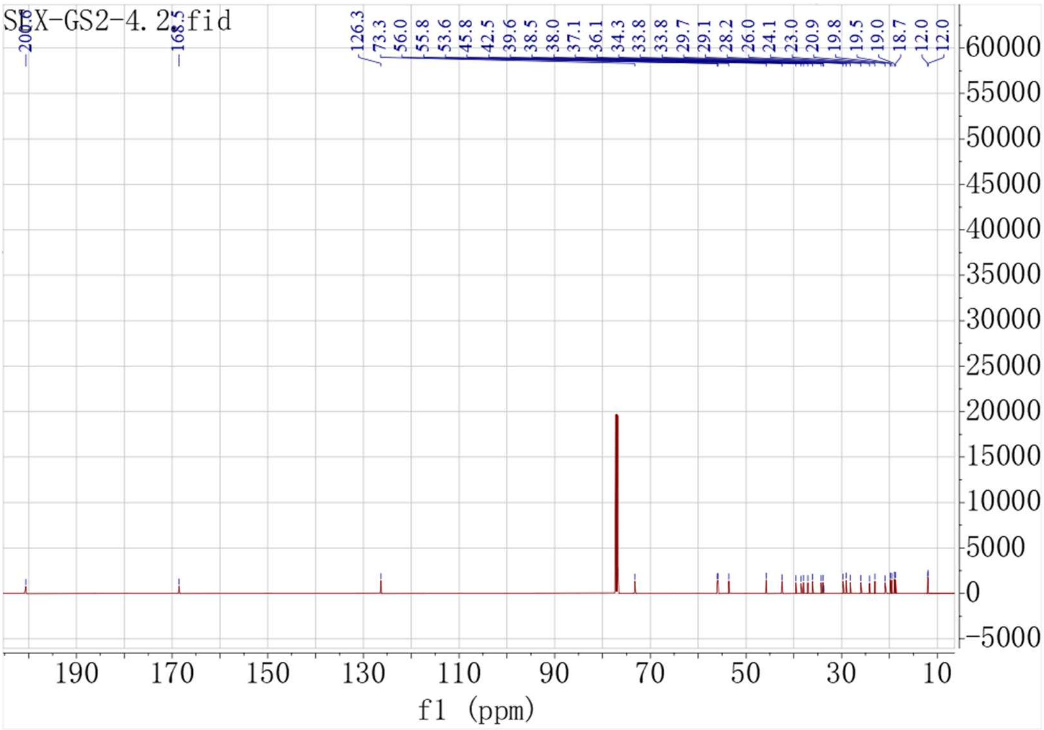

Figure S4. ^{13}C-NMR spectrum of **2** ($CDCl_3$, 150MHz)

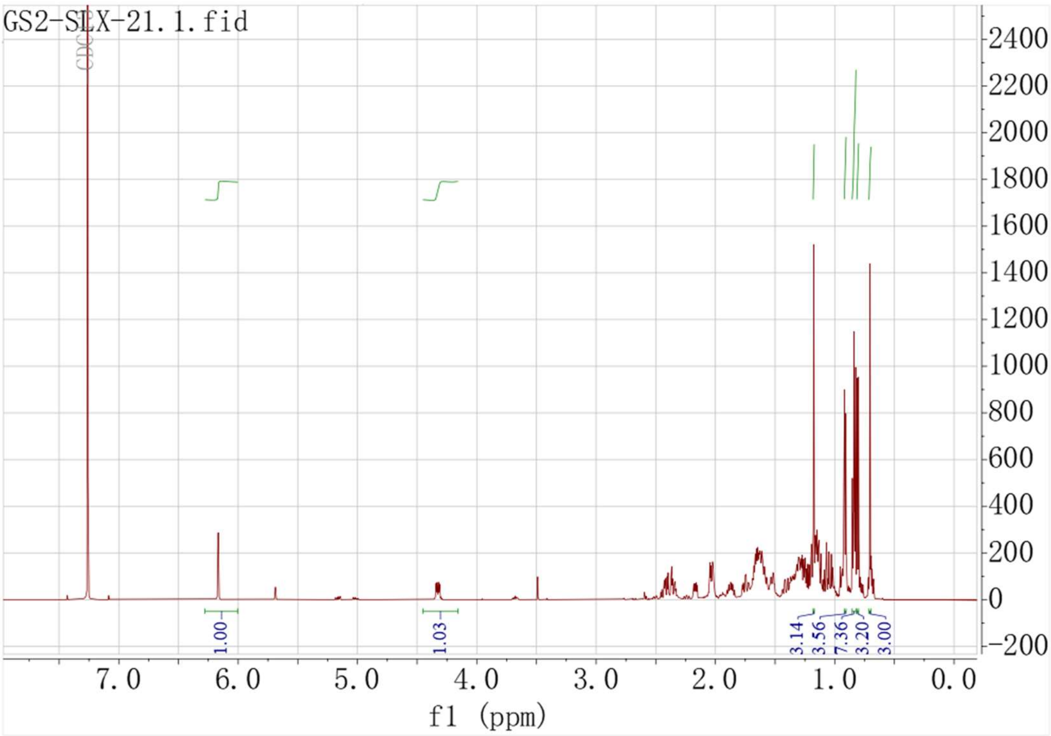

Figure S5. ^{1}H-NMR spectrum of **3** ($CDCl_3$, 600MHz)

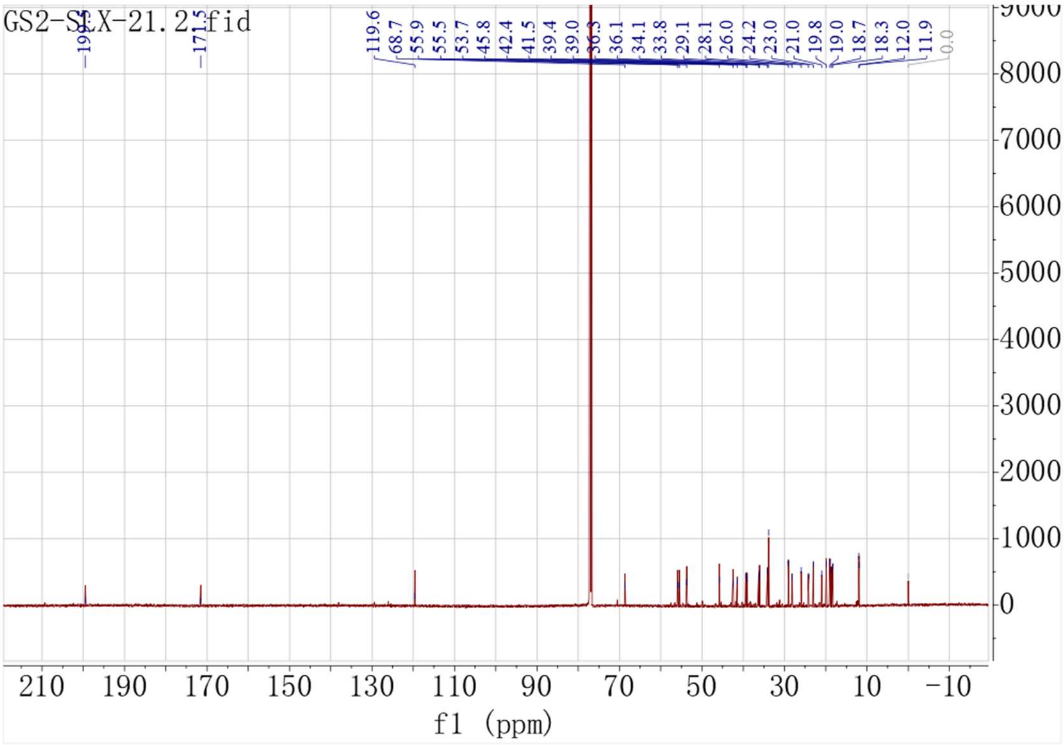

Figure S6. ^{13}C-NMR spectrum of **3** ($CDCl_3$, 150MHz)

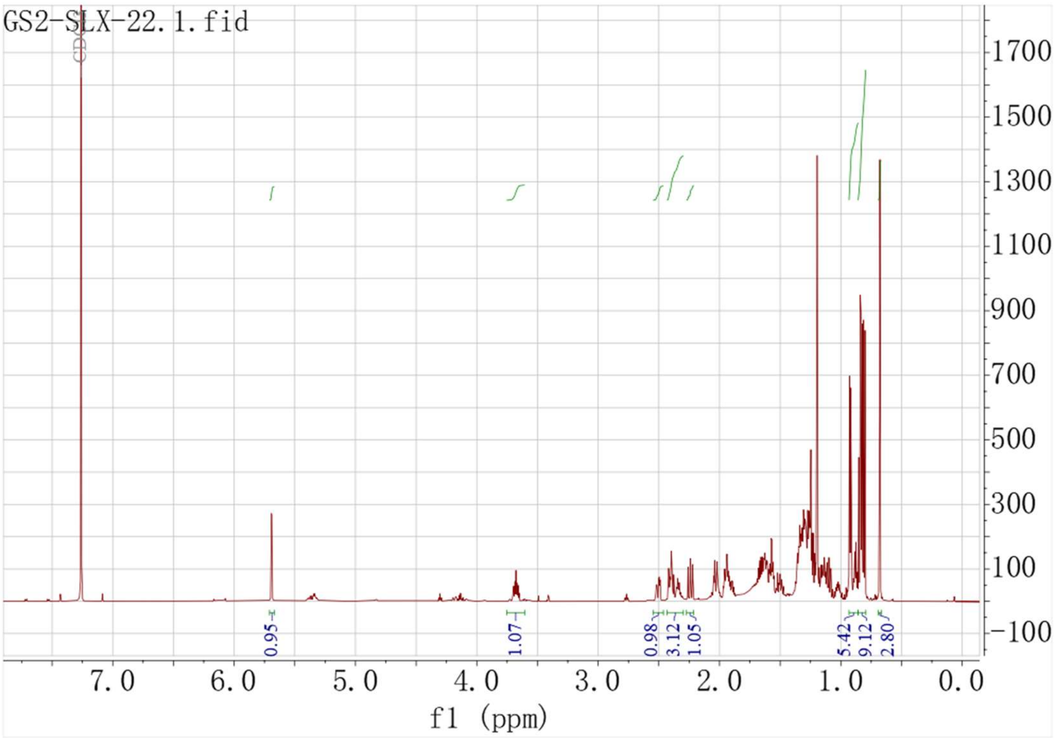

Figure S7. ^{1}H-NMR spectrum of **4** ($CDCl_3$, 600MHz)

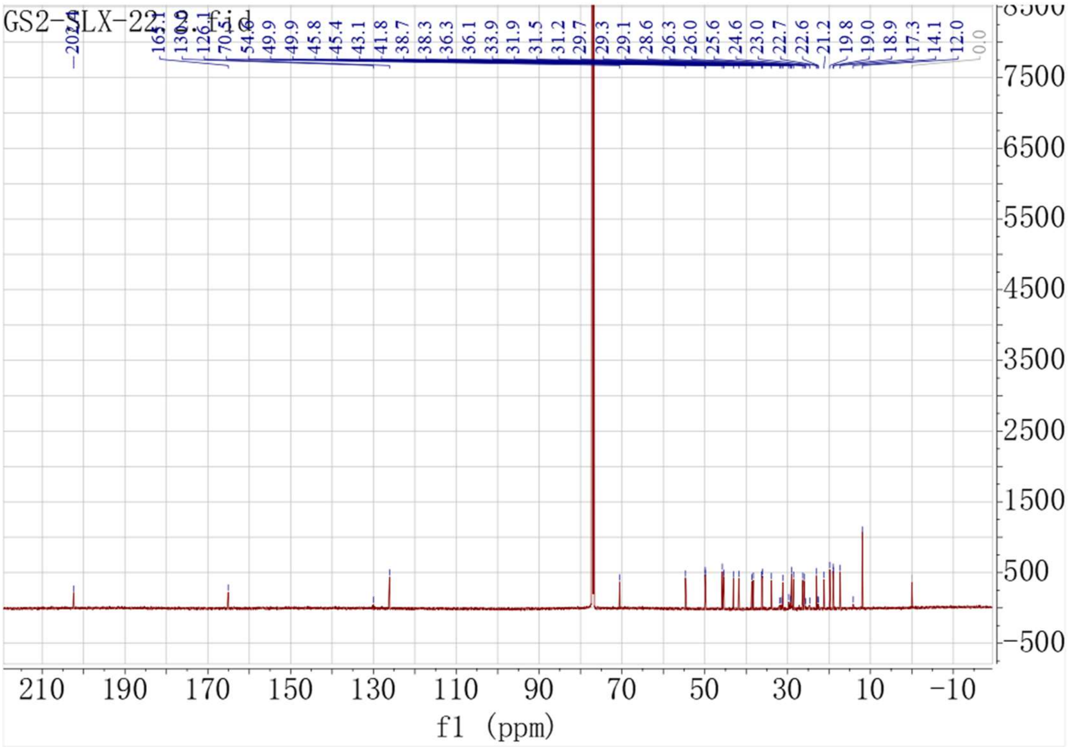

Figure S8. ^{13}C-NMR spectrum of **4** ($CDCl_3$, 150MHz)

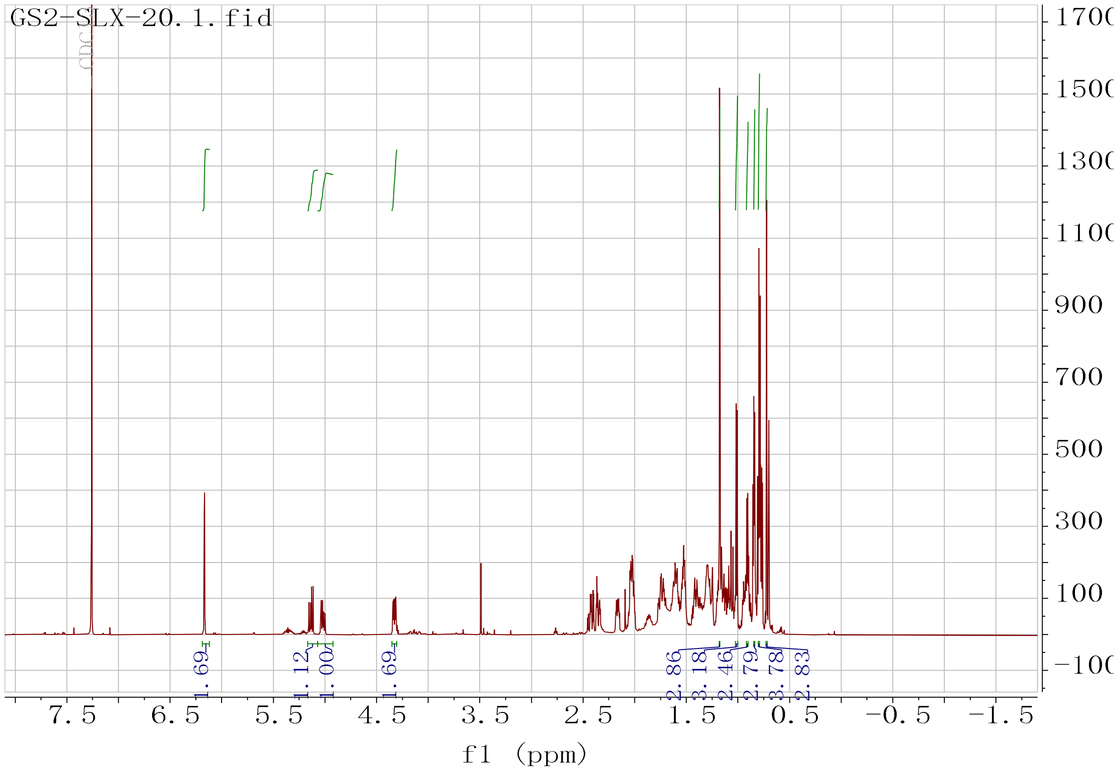

Figure S9. ^{1}H-NMR spectrum of **5** ($CDCl_3$, 600MHz)

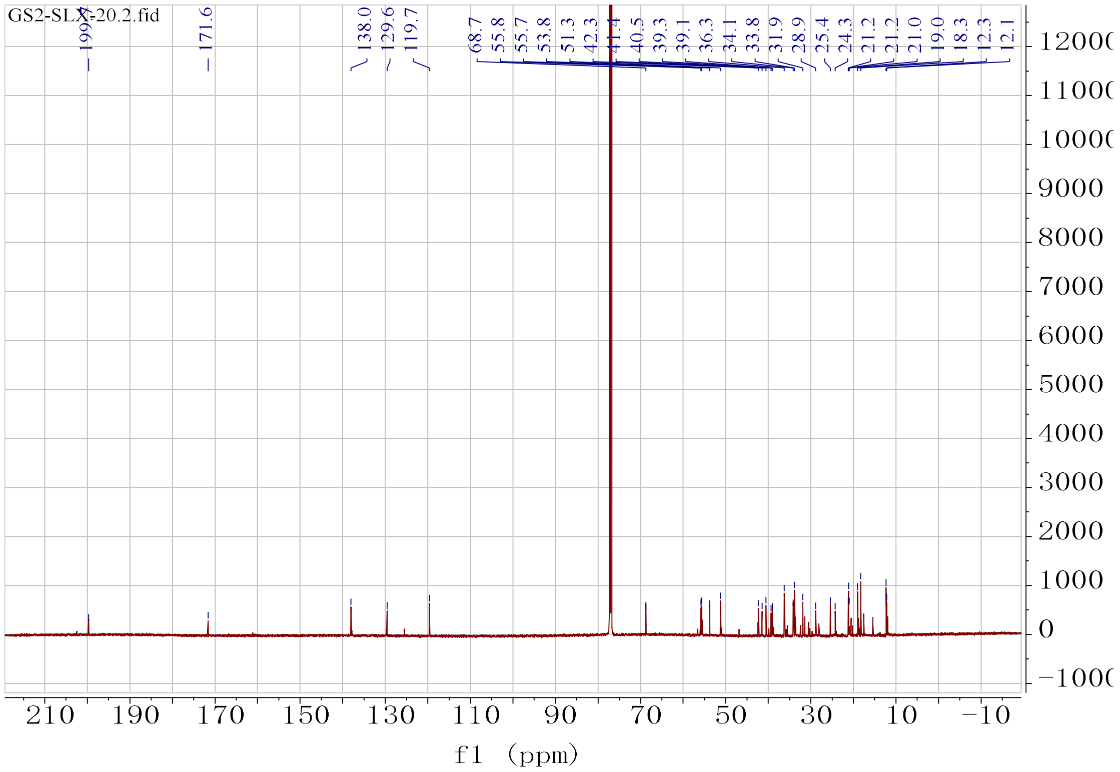

Figure S10. ^{13}C-NMR spectrum of **5** ($CDCl_3$, 150MHz)

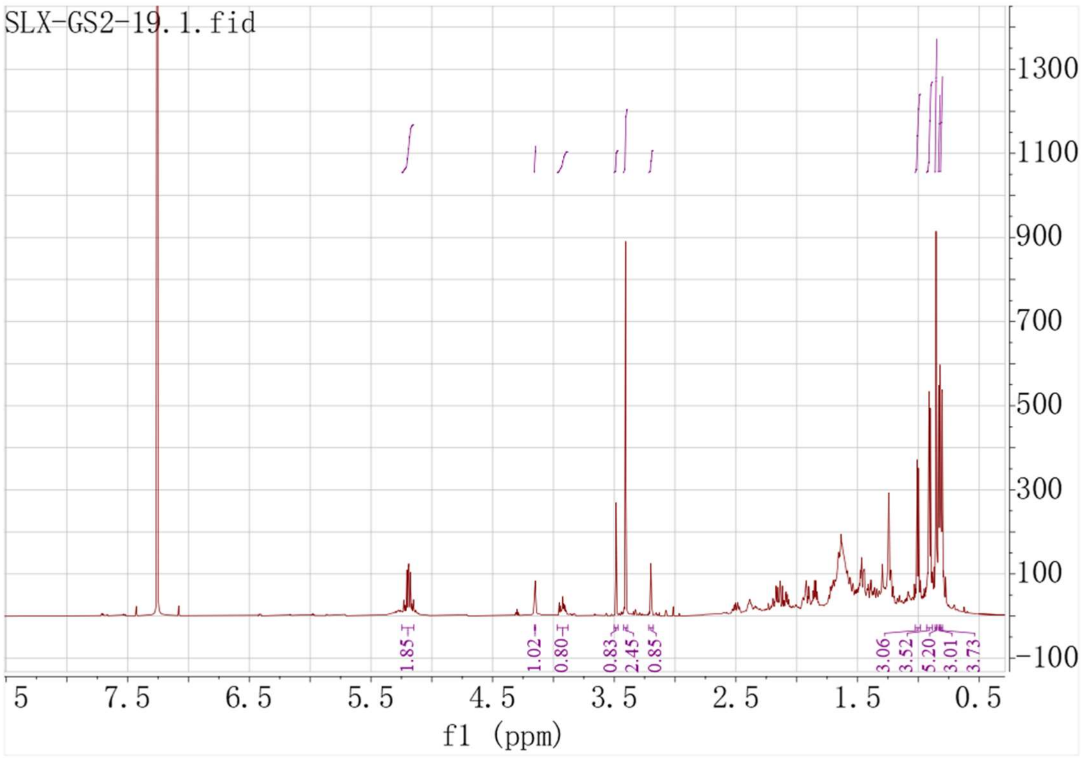

Figure S11. ^{1}H-NMR spectrum of **6** ($CDCl_3$, 600MHz)

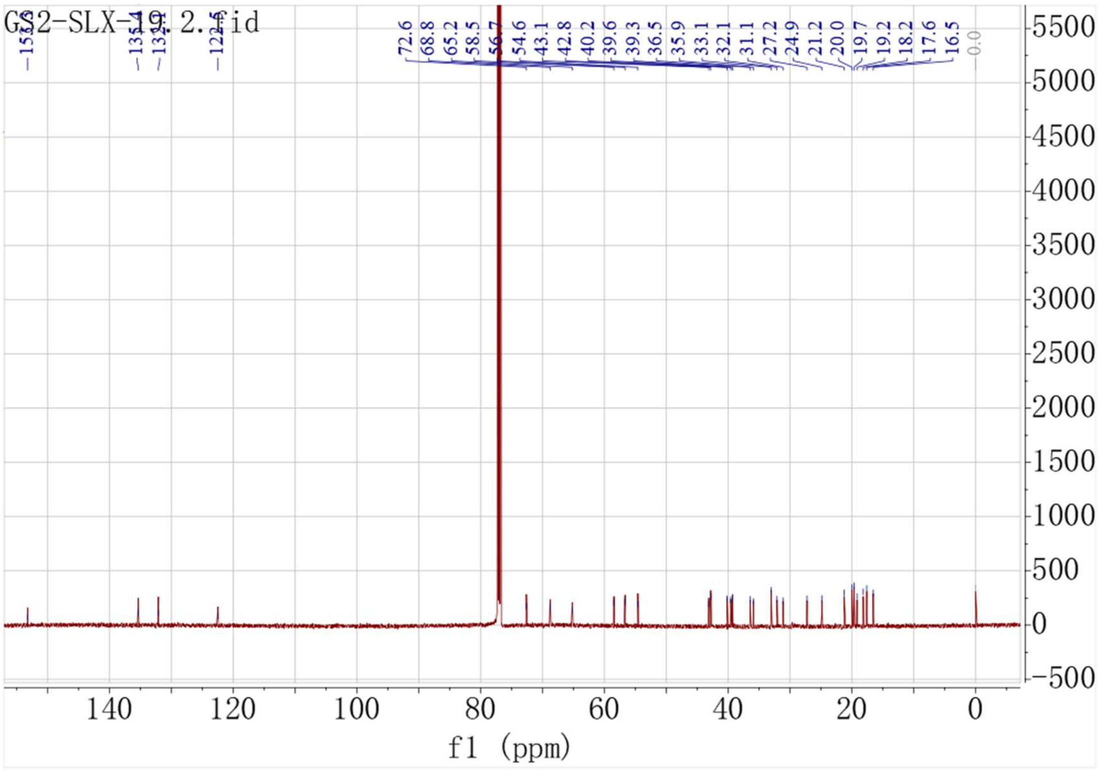

Figure S12. ^{13}C-NMR spectrum of **6** ($CDCl_3$, 150MHz)

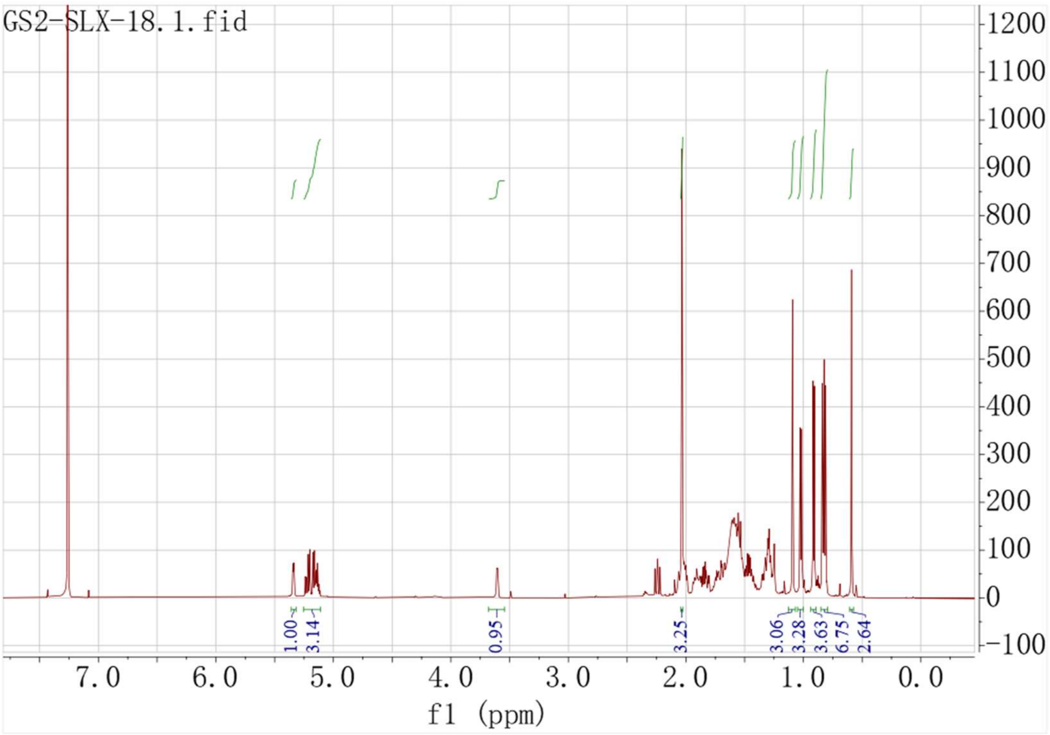

Figure S13. ^{1}H-NMR spectrum of **7** (CDCl$_3$, 600MHz)

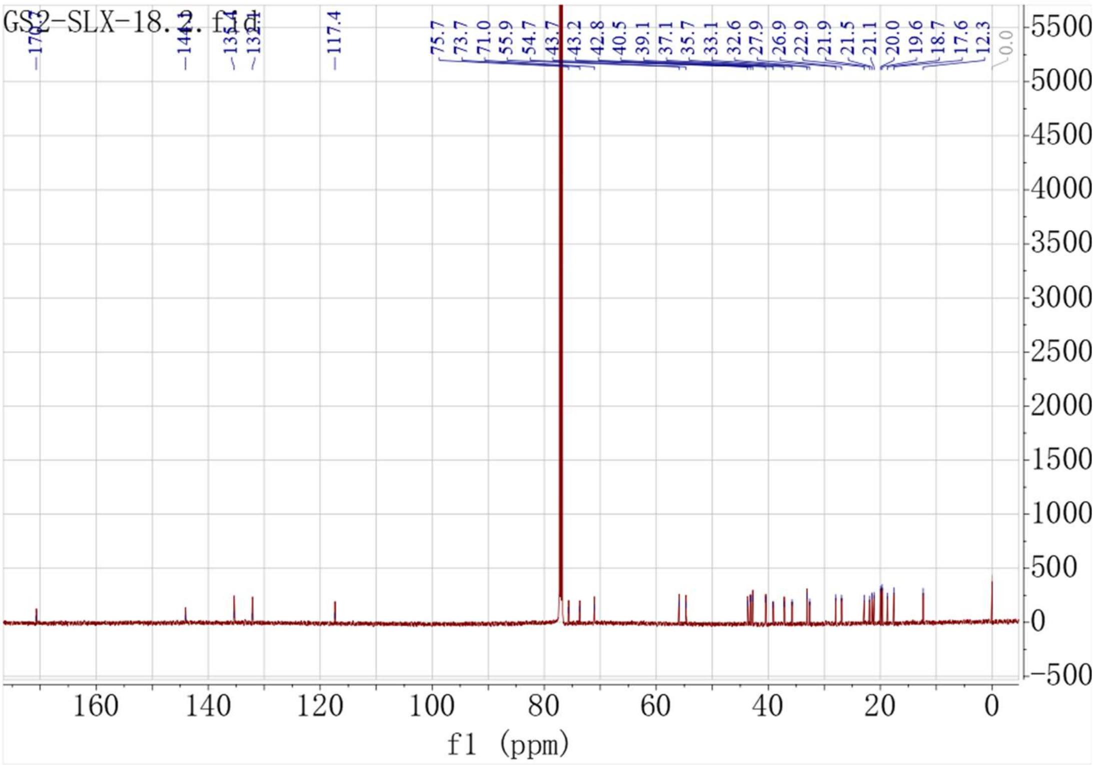

Figure S14. ^{13}C-NMR spectrum of **7** (CDCl$_3$, 150MHz)

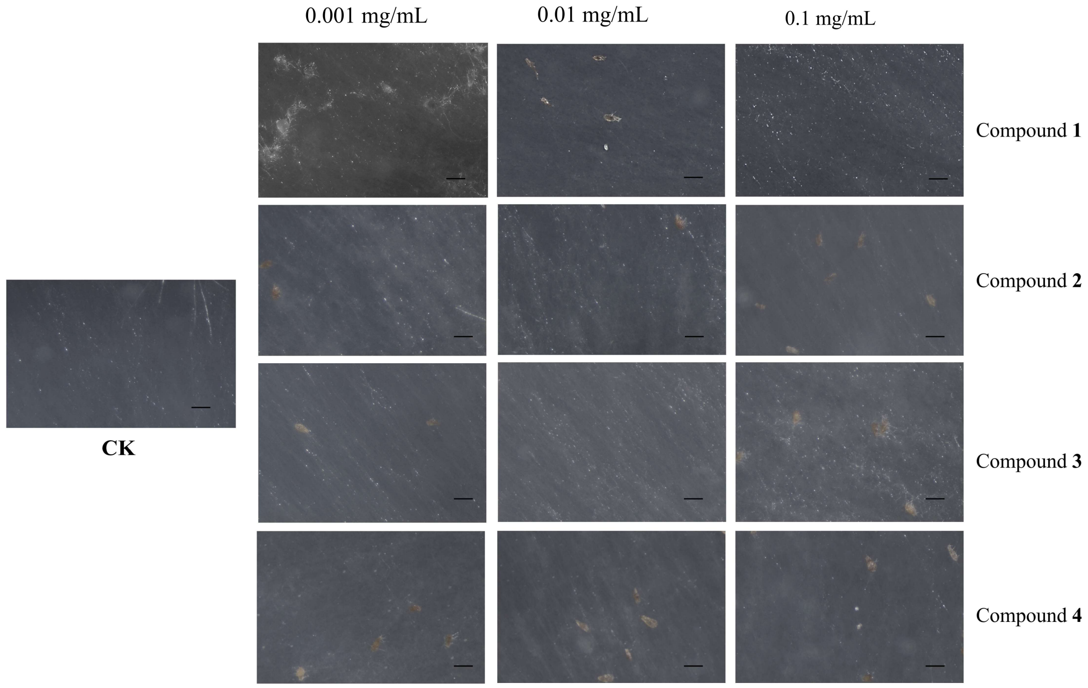

Figure S15. Growth characteristics of *Ceratobasidium* GS2 in control group and compounds **1-4** treatment. Scale bars=500 μm

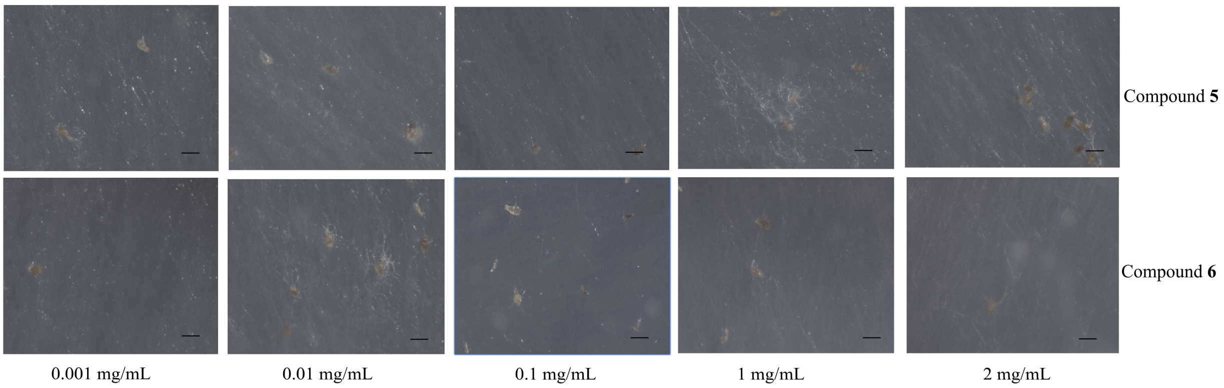

Figure S16. Growth characteristics of *Ceratobasidium* GS2 in compounds **5-6** treatment. Scale bars=500 μm
